# Supplementary material for: Long-Term Satisfaction and Patient-Centered Outcomes of Deep Brain Stimulation in Parkinson’s Disease
Source: Brain Sci. 2018 Apr 1;8(4):60. doi: 10.3390/brainsci8040060 (PMC5924396; doi:10.3390/brainsci8040060)
Supplement: Supplementary file 1 [file brainsci-08-00060-s001.pdf]

Table S1. Correlation of Change in Reported Symptom and Satisfaction

Symptom	Spearman Correlation Coefficient	p-value
Dyskinesia	0.22	0.13
Motor fluctuations	0.27	0.051
Tremor	0.20	0.16
Rigidity	0.29	0.04
Bradykinesia	0.30	0.03
Gait difficulties	0.26	0.07
Balance, falling	0.12	0.41
Off-period dystonia	0.21	0.13
Cognitive impairment	0.09	0.54
Depression	0.38	0.01
Apathy	0.40	0.003
Insomnia	0.43	0.001
Excessive daytime sleepiness	0.16	0.27
Pain	0.37	0.01
Medication side effects	0.13	0.37

Correlation of change in symptoms from pre-DBS to current time (8.2±2.6 years post DBS) with current patient satisfaction. Worsened severity in symptoms correlated with lower satisfaction.

Table S2. Patient Reported Symptom Severity

Symptom	n (%)
Dyskinesia	
Better, n(%)	38 (73.1)
Did not change, n(%)	10 (19.2)
Worse, n(%)	4 (7.7)
Motor fluctuations	
Better	42 (80.8)
Did not change	7 (13.5)
Worse	3 (5.8)
Tremor	
Better	41 (78.9)
Did not change	9 (17.3)
Worse	2 (3.9)
Rigidity	
Better	33 (63.5)
Did not change	10 (19.2)
Worse	9 (17.3)
Bradykinesia	
Better	24 (46.2)
Did not change	12 (23.1)

Worse	16 (30.8)
Gait difficulties	
Better	23 (44.2)
Did not change	3 (5.8)
Worse	26 (50)
Balance, falling	
Better	13 (25)
Did not change	5 (9.6)
Worse	34 (65.4)
Off-period dystonia	
Better	19 (36.5)
Did not change	17 (32.7)
Worse	16 (30.8)
Cognitive impairment	
Better	7 (13.5)
Did not change	18 (34.6)
Worse	27 (51.9)
Depression	
Better	13 (25)
Did not change	22 (42.3)
Worse	17 (32.7)
Apathy	
Better	13 (25)
Did not change	23 (44.2)
Worse	16 (30.8)
Insomnia	
Better	16 (30.8)
Did not change	17 (32.7)
Worse	19 (36.5)
Excessive daytime sleepiness	
Better	10 (19.2)
Did not change	20 (38.5)
Worse	22 (42.3)
Pain	
Better	16 (30.8)
Did not change	20 (38.5)
Worse	16 (30.8)
Medication side-effects	
Better	27 (51.9)
Did not change	20 (38.5)
Worse	5 (9.6)

Patients reported improvement, no change and worsening of motor and non-motor symptoms at two time points (pre-operatively and at the current time of 8.2±2.6 years post DBS).

Table S3. Locally developed DBS patient centered outcome questionnaire (RUSH-DBS-Q)

Symptoms	Spearman correlation coefficient	p-value
Dyskinesia	0.22	0.13
Motor fluctuations	0.27	0.051
Tremor	0.20	0.16
Rigidity	0.29	0.04
Bradykinesia	0.30	0.03
Gait difficulties	0.26	0.07
Balance, falling	0.12	0.41
Off-period dystonia	0.21	0.13
Cognitive impairment	0.09	0.54
Depression	0.38	0.01
Apathy	0.40	0.003
Insomnia	0.43	0.001
Excessive daytime sleepiness	0.16	0.27
Pain	0.37	0.01
Medication side-effects	0.13	0.37

Values represent Spearman correlation coefficients. Correlation of change in symptoms from pre-DBS to current time (8.2±2.6 years post DBS) with current patient satisfaction. Worsened severity in symptoms correlated with lower satisfaction.

Table S4. Correlation of Baseline Motor Scores and Satisfaction

UPDRS-III Pre-DBS sub-scales	Spearman Correlation Coefficient	p-value
UPDRS-III-axial-ON	0.14	0.33
UPDRS-III-axial-OFF	0.08	0.59
UPDRS-III-tremor-resting-ON	-0.11	0.46
UPDRS-III-tremor-resting-OFF	-0.06	0.68
UPDRS-III-tremor-postural-ON	-0.15	0.3
UPDRS-III-tremor-postural-OFF	0.09	0.54
UPDR-III-bradykinesia-left-ON	0.01	0.95
UPDRS-III-bradykinesia-left-OFF	-0.14	0.33
UPDRS-III-bradykinesia-right-ON	-0.06	0.69
UPDRS-III-bradykinesia-right-OFF	-0.12	0.4
UPDRS-III-rigidity-ON	-0.02	0.87
UPDRS-III-rigidity-OFF	0.02	0.9

Correlation of baseline UPDRS-III sub-scale scores and current patient satisfaction.

Table S5. Correlation of Reported Symptom Severity and Satisfaction.

Symptom	Spearman Correlation Coefficient	p-value
Motor symptoms	0.36	0.01
Non-motor symptoms	0.33	0.02

Patients' reported total motor and non-motor scores (change from pre-DBS to current time of 8.2±2.6 years post DBS) from the RUSH-DBS-Q were correlated to patient satisfaction.
